# Arrays of Si vacancies in 4*H*-SiC produced by focused Li ion beam implantation

**DOI:** 10.1038/s41598-021-82832-x

**Published:** 2021-02-11

**Authors:** Shojan P. Pavunny, Andrew L. Yeats, Hunter B. Banks, Edward Bielejec, Rachael L. Myers-Ward, Matthew T. DeJarld, Allan S. Bracker, D. Kurt Gaskill, Samuel G. Carter

**Affiliations:** 1 U. S. Naval Research Laboratory, 4555 Overlook Ave. SW, Washington, DC 20375 USA; 2 Sandia National Laboratories, Albuquerque, NM 87185 USA; 3 Present Address: Institute for Research in Electronics and Applied Physics, University of Maryland, College Park, MD 20742 USA

**Keywords:** Semiconductors, Quantum optics, Single photons and quantum effects, Condensed-matter physics

## Abstract

Point defects in SiC are an attractive platform for quantum information and sensing applications because they provide relatively long spin coherence times, optical spin initialization, and spin-dependent fluorescence readout in a fabrication-friendly semiconductor. The ability to precisely place these defects at the optimal location in a host material with nano-scale accuracy is desirable for integration of these quantum systems with traditional electronic and photonic structures. Here, we demonstrate the precise spatial patterning of arrays of silicon vacancy (${V}_{Si}$) emitters in an epitaxial *4H*-SiC (0001) layer through mask-less focused ion beam implantation of Li^+^. We characterize these arrays with high-resolution scanning confocal fluorescence microscopy on the Si-face, observing sharp emission lines primarily coming from the ${V1}^{{\prime}}$ zero-phonon line (ZPL). The implantation dose is varied over 3 orders of magnitude, leading to ${V}_{Si}$ densities from a few per implantation spot to thousands per spot, with a linear dependence between ZPL emission and implantation dose. Optically-detected magnetic resonance (ODMR) is also performed, confirming the presence of *V*2 ${V}_{Si}$. Our investigation reveals scalable and reproducible defect generation.

## Introduction

Quantum information science and technology is of great interest for fundamental advances in computing, sensing, and communication. Defects in wide bandgap solids are promising as a solid-state implementation of quantum bits (qubits) since the defects often host spins with long coherence times, are optically active, and are straightforward to produce in ensembles or at the single defect limit^1,2^. The spin states can often be optically initialized with laser light, read-out through spin-dependent photoluminescence, and even have a spin-photon interface^3–9^. Though nitrogen-vacancy (N-*V*) color centers in diamond have emerged as a “gold standard” for defect spin qubits, limitations like immature device fabrication protocols, material cost, availability and non-ideal optical properties^10–12^ have led to considerable interest in other potential qubit platforms. Intra-bandgap atomic-scale point defects in the CMOS device friendly opto(electronic) material SiC^13,14^ are promising because of their bright and photo-stable emission, the low abundance of nuclear spins, and, in some cases, long spin coherence times of 100 µs or more^2,6,15–17^. A number of different defects have been shown to have long-lived spin states in this polymorphic material system: silicon vacancies (${V}_{Si}$), divacancies (${V}_{C}{V}_{Si}$), and N-*V* centers^15,16,18–20^. The ${V}_{Si}$ has been of particular interest as it has demonstrated long, room temperature spin coherence (spin-echo T_2_ of ~ 100 μs), sharp ZPLs, and a high spin system (S = 3/2)^8,9,15,17,19,21,22^.

The ability to precisely create the desired ${V}_{Si}$ density at the optimal location in a semiconductor host with nanometer accuracy is highly desirable for applications in scalable quantum information processing and sensing^23–27^. A number of techniques have been used to reach this goal. In Ref. 23, a ~ 1 µm proton beam was used for implantation with optical alignment, and in Ref. 25 an electron beam lithography mask of ~ 65 nm holes was used with broad beam implantation of carbon ions. Here, we demonstrate the precise generation of ${V}_{Si}$ emitter arrays in an epitaxial 4*H*-SiC layer through focused ion implantation^28^, a reliable, versatile, repeatable, and CMOS compatible top-down approach. This technique enables the implantation of different species, isotopes, and energies of ions. When combined with either counted or timed processes, various doses can be implanted in different areas of the same sample without introducing multiple masking steps or multiple samples. Generation of ${V}_{Si}$ in 4*H*-SiC has previously been demonstrated using focused ion implantion^24^ of Si^2+^. Lithium ions are chosen here as they are the lightest ion delivered by liquid metal alloy ion sources (LMAIS), which gives them a low energy spread (∝ *m*^0.33^, where, *m* is the ion mass) and high spatial resolution^29^. We use Sandia’s nanoImplanter (nI), a 10–200 keV focused ion beam machine (A&D FIB100nI), with a < 20 nm spot size. Previous work implanting Si ions in diamond using this nI achieved high alignment resolution of < 50 nm and improved coupling strength between the implanted emitter and the photonic structure^30^.

## Approach

In order to ensure a low concentration of native defects, a high quality SiC layer of ~ 10 µm thickness is homoepitaxially grown on Si polar face of (0001) n + 4*H*-SiC substrate (II–VI, Inc.) oriented 4° off the *c*-axis by hot-wall chemical vapor deposition. Under optimized growth conditions yielding low epitaxial defects, the nitrogen flow is controlled to have a low n-type conductivity of 5 × 10^14^ cm^−3^, and the epilayer is characterized as described elsewhere^31^. The Si-face of the epilayer sample (~ 1 × 1 cm^2^) with lithographically patterned alignment marks is subsequently implanted along the growth axis, employing mass-filtered Li^+^ ions from LMAIS, AuSiLi. Room-temperature implantation is performed in the form of 10 × 10 square matrix arrays of spots (a cell of area ~ 50 × 50 µm^2^ with a spot pitch of ~ 5 µm) using one of the nine different fixed doses in the range of 10–6000 ions/spot (2.01 × 10^12^–1.20 × 10^15^ ions cm^−2^) on a particular cell. The lowest dose is chosen to generate single or few ${V}_{Si}$ emitters as the expected implantation yield is about 4–10% based on previous studies using high energy proton irradiation and low energy Si^2+^ implantation^23,24,27^. The difference in the range of yields is likely due to the ion range and the likelihood of isolated ${V}_{Si}$ formation relative to large scale damage clusters at the ion end-of-range. The highest dose employed in the scheme is well below the critical dose, ~ 10^16^ ions cm^−2^, of SiC to avoid amorphization^32^. With the 100 keV energy used in this work, the ion implantation depth in the Z-direction and the lateral projection in the X–Y plane are estimated at 370 ± 70 nm and ± 70 nm, respectively, using Stopping and Range of Ions in Matter (SRIM) software allowing the formation of defects at a significant depth within the semiconductor (See Fig. S1 in the Supplemental Material).

## Results and discussion

**Figure 1:**
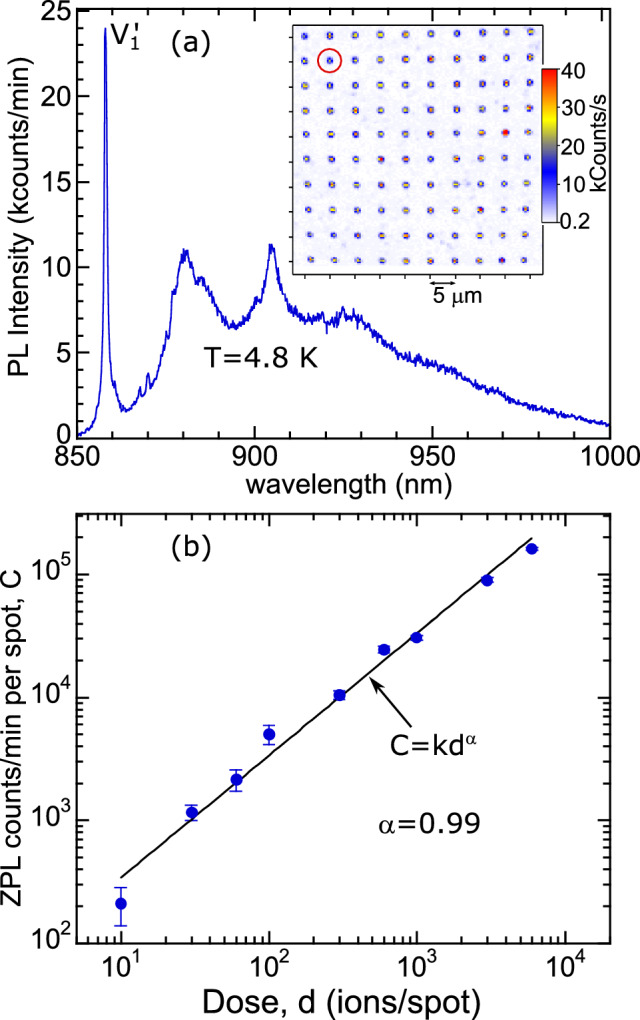
(**a**) Background subtracted PL spectrum at 4.8 K acquired from an individual spot of a 10 × 10 array implanted at a dose of 1.2 × 10^14^ ions/cm^2^ (600 ions/spot) which is identified (red circle) in the LT confocal fluorescence map (inset). (**b**) Mean ${V}_{1}^{{\prime}}$ ZPL counts (circles) as a function of Li^+^ implantation dose, along with a fit to a power law, $C=k{d}^{\alpha }$. Error bars represent twice the standard error of the mean.

We carried out low temperature (LT) as well as room temperature (RT) confocal integrated fluorescence maps of cells having implantation doses of 10, 30, 60, 100, 300, 600, 1000, 3000, and 6000 ions/spot in order to locate the emitter arrays in the sample. The inset of Fig. 1a shows a typical LT map of the patterned 10 × 10 array of isolated spots, having the same pitch as that of the implanted pattern, created by an intermediate dose of 600 ions/spot. Figure 1a depicts the background-subtracted PL spectrum collected in the 850–1000 nm wavelength range at 4.8 K on one (circled) of the 100 implantation spots identified in the above map. The background spectrum is collected from a non-implanted region between two spots.

The PL spectrum includes a sharp ZPL at ~ 858.2 nm (~ 1445 meV) with a full width at half maximum (FWHM) of ~ 1.2 nm along with a broad phonon sideband (PSB). Similar spectra are consistently observed at the implanted spots, with the intensity changing for the different doses. We attribute this emission to the ${V}_{Si}$, with the observed ZPL corresponding to the ${V1}^{{\prime}}$ line^33,34^. There are two distinct types of ${V}_{Si}$ in 4H-SiC, labeled $V1$ and $V2$, that correspond to the two inequivalent lattice sites for a point defect in that material^33–36^. Both have characteristic PL spectra that have been previously identified, and which are most easily distinguished by the appearance of their sharp ZPLs at LT^33,35^. The $V1$ lattice defect has two possible ZPLs, $V1$ (~ 862 nm) and ${V1}^{{\prime}}$ (~ 859 nm), corresponding to its ^4^*A*_2_ and ^4^*E* excited states, respectively^34,37^. The dipole moment of $V1$ (${V1}^{{\prime}}$) is parallel (perpendicular) to the *c*-axis, so emission from the sample face is weak (strong). The relative emission strength of $V1$ and ${V1}^{{\prime}}$ has also shown a dependence on temperature and excitation power in previous studies^33,38^. $V2$ only has one ZPL (~ 916 nm), and its emission is also weak along the *c*-axis, due to its parallel dipole moment^34,37^. Strong emission from ${V1}^{{\prime}}$ and weaker emission from $V1$ and $V2$ ZPLs has been observed previously in this geometry^24,25^. In the present experiments, only the ZPL of ${V1}^{{\prime}}$ is reliably observed at implantation sites, consistent with near *c*-axis collection and the dipole moments involved. In some spectra weak emission from the $V1$ and $V2$ ZPLs is observed. Later, we also show RT ODMR from implanted sites with a clear signature of *V*2, implying that *V*2 must emit to some degree in the PSB even when collecting along the *c*-axis. Therefore, we expect PSB emission is present from *V*1 and $V2$. The spectra we observe confirm that silicon vacancies are formed, and the array clearly demonstrates the usefulness of Li^+^ implantation approach.

The average brightness of the implanted spots is plotted as a function of dose in Fig. 1b for ~ 1 mW (~ 2 × 10^9^ W m^−2^) excitation. The average count rates ($C$) of ${V1}^{{\prime}}$ ZPL at LT are measured for the first 20 spots in each cell of dose ($d$) 30–6000 ions/spot and for the first 50 spots in the cell of dose 10 ions/spot. We find that brightness scales linearly with implantation dose all the way up to the highest implantation dose of 6000 ions/spot. A power law fit of $C=k{d}^{\alpha }$ yields $\alpha$ = 0.99 ± 0.04. This result is in contrast to those from focused ion beam implantation of 35 keV Si^2+^, which started showing saturation from 100 to 700 ions/spot^24^. In part, this may be due to the much shallower stopping depth and the larger number of vacancies created for 35 keV Si^2+^ (18.5 nm and ~ 0.7 vacancies per Angstrom per ion, respectively) compared to 100 keV Li^+^ (370 nm and ~ 0.045 vacancies per Angstrom per ion, respectively). This results in a significantly higher density of vacancies and damage to the lattice in the Si case as compared to the Li implantation.

**Figure 2:**
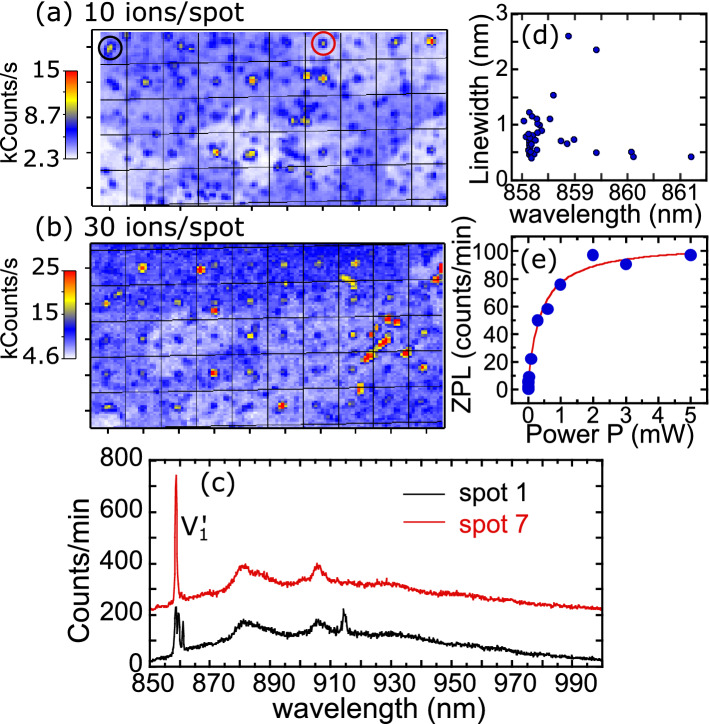
(**a**, **b**) LT confocal fluorescence maps integrating over the entire spectrum at the two lowest implantation doses: (**a**) 10 ions/spot and (**b**) 30 ions/spot. A grid is superimposed on the image as a guide to the eye. The expected implantation site is at the center of each grid square. (**c**) LT PL spectra from two spots of the 10 ions/spot array, with the spot 7 spectrum offset by 200 counts/min. Spots 1 and 7 are identified in (**a**) with black and red circles, respectively. (**d**) ${V1}^{{\prime}}$ ZPL linewidth at 4.8 K versus center emission wavelength for the 10 ions/spot array. (**e**) ${V1}^{{\prime}}$ ZPL saturation curve at LT.

In Fig. 2a,b we present LT confocal fluorescence maps of 50 spots for cells implanted with the lowest doses of 10 and 30 ions/spot, respectively. A grid is superimposed on the map as a guide to the eye, with implanted spots expected near the center of each grid square. For the 30 ions/spot dose, the pattern of bright spots is fairly clear, although there is a significant background of emission spots not associated with the implantation pattern. For the 10 ions/spot dose, the implantation pattern is more difficult to see, due to background emission spots and the lack of emission from some implantation sites. ${V1}^{{\prime}}$ ZPL is detected on all implanted spots for Li-ion doses of 30 ions/spot or higher. At the lowest dose of 10 ions/spot, 37 of the 50 spots show ${V}_{Si}$ emission. Typical LT PL spectra from two of these spots (circled in Fig. 2a) are shown in Fig. 2c. The spectrum for spot 7 is relatively simple, showing the ${V1}^{{\prime}}$ ZPL with a linewidth (FWHM) of ~ 0.6 nm along with the broad PSB. The spectrum for spot 1 has more features, showing multiple lines near 860 nm and a line at 915 nm, corresponding to *V*2. This likely indicates multiple ${V}_{Si}$ with some variation in the ZPL. To better characterize the variation in the spectrum from one implantation spot to another, Fig. 2d plots the ${V1}^{{\prime}}$ ZPL linewidth vs. center wavelength (λ_0_) at 4.8 K for the 37 identified emitting spots. While 70% of the spots emit at ~ 858.2 ± 0.1 nm with a narrow FWHM of ~ 0.74 ± 0.21 nm, 30% of them showed a spectral drift as large as ~ 3 nm towards longer wavelengths with λ_0_ ~ 859.4 ± 0.8 nm and FWHM ~ 0.92 ± 0.78 nm. The observed red shift in λ_0_ may be due to strain at the implanted spot or perhaps due to the presence of implanted Li ions.

The measurements presented so far were performed at an excitation power near saturation. Figure 2e plots the intensity ($$I$$) of $${V1}^{{\prime}}$$ ZPL as a function of pump power ($$P$$, 1–5000 µW) from the background corrected LT spectra at the 10 ions/spot dose. From a fit to the function $$I={I}_{S}/\left[1+\left(\frac{{P}_{S}}{P}\right)\right]$$, the saturation intensity $${I}_{S}$$ and the saturation power ($${P}_{S})$$ are estimated to be ~ 105 ± 4 counts/min and ~ 380 ± 58 µW, respectively. Additional measurements of the emission stability, temperature dependence, and excitation wavelength dependence can be found in the Supplemental Material.

**Figure 3:**
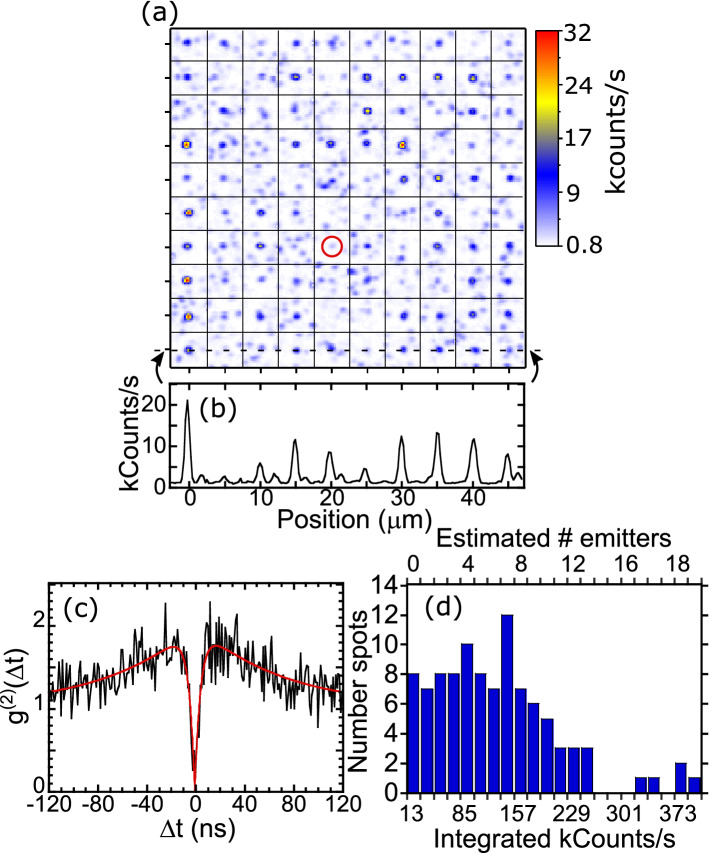
(**a**) Room temperature confocal fluorescence map of a 10 ions/spot pattern after further cleaning and 400 °C anneal. Tick marks represent 5 µm. (**b**) Line cut of integrated emission counts vs. position. (**c**) Background-corrected second-order autocorrelation function ${g}^{\left(2\right)}\left(\Delta t\right)$ at room temperature from the spot circled in (**a**). (**d**) Histogram of emission counts for the 50 spots in the implantation pattern in (**a**).

In an attempt to improve the ${V}_{Si}$ emission brightness and reduce background emission, we cleaned the sample and annealed at 400 °C for 2 h. This anneal temperature is based on improvements shown in other studies, while keeping the temperature low enough to avoid converting ${V}_{Si}$ into other defects^39–41^. The cleaning consisted of Acetone/Isopropanol at 40 °C, Piranha (5:1 H_2_SO_4_:H_2_O_2_ at 100 °C), 5:1:1 NH_4_OH:H_2_O_2_:H_2_O at 40 °C, and a 4 h soak in 1:1 49% HF:69% HNO_3_. Measuring at RT with a higher 0.9 NA objective also made a difference. Figure 3a displays a RT confocal fluorescence map of a 10 ions/spot array. The implantation pattern is significantly easier to see than in Fig. 2a although there are still many background emission spots not associated with pattern. In Fig. 3b, we display a line cut through the last row of the pattern, which clearly shows emission at almost every multiple of 5 µm, with a few peaks off of the pattern.

Hanbury Brown–Twiss (HBT) interferometry^42^ measurements are performed on a few of the spots to examine their non-classical emission properties and check for the existence of single ${V}_{Si}$ centers. Figure 3c shows the background corrected second-order autocorrelation function ${g}^{\left(2\right)}(\Delta t)$ measured on the red circled spot in Fig. 3a. The signal count $s$ for this spot is about 2100 counts/s, with a background $b$ of about 1300 counts/s. The background corrected function is given by^25^ ${g}_{\text{corr}}^{\left(2\right)}\left(\Delta t\right)=\left[{g}_{raw}^{2}\left(\Delta t\right)-\left(1-{\rho }^{2}\right)\right]/{\rho }^{2}$, where $\rho =\frac{s}{s+b}=0.618$. The data goes to nearly zero at $\Delta t=0$, indicating a single emitter at this spot. The data are fit using the relation^43^ ${g}^{\left(2\right)}(\Delta t)=1-\beta {e}^{-\left|\frac{\Delta t}{{\tau }_{1}}\right|}-(1-\beta ){e}^{-\left|\frac{\Delta t}{{\tau }_{2}}\right|}$, where ${\tau }_{1}$ and ${\tau }_{2}$ are times associated with the radiative lifetime and intersystem crossing of the defect, respectively, and $\beta$ is related to the shelving fraction. We obtain $\beta =2.0\pm 0.12$, ${\tau }_{1}=4.7\pm 0.9$ ns, and ${\tau }_{2}=72\pm 10$ ns, consistent with previous ${V}_{Si}$ measurements^25,44^. By summing pixels in Fig. 3a in a 1.75 × 1.75 µm square around each site and subtracting background counts, we obtain the histogram plotted in Fig. 3d. (Note that a peak count rate of 8000 counts/s gives a spatially integrated count rate in the histogram of about 100,000 counts/s.) The bin size is chosen to place the circled spot in the second bin, corresponding to one emitter. The histogram is affected by the background emission spots but should still provide reasonable estimates on the number of emitters. Based on the average count rate, the mean number of emitters per spot is about 6. This corresponds to a conversion yield of ~ 60%, significantly higher than obtained from other studies using high energy protons (10%)^23^, low energy carbon ions (19%)^25^, and low energy silicon ions (3.9%)^24^. The high yield may be related to a combination of producing defects further below the surface^45^ than higher mass/lower energy ions and a higher vacancy creation probability per depth than high energy protons.

**Figure 4:**
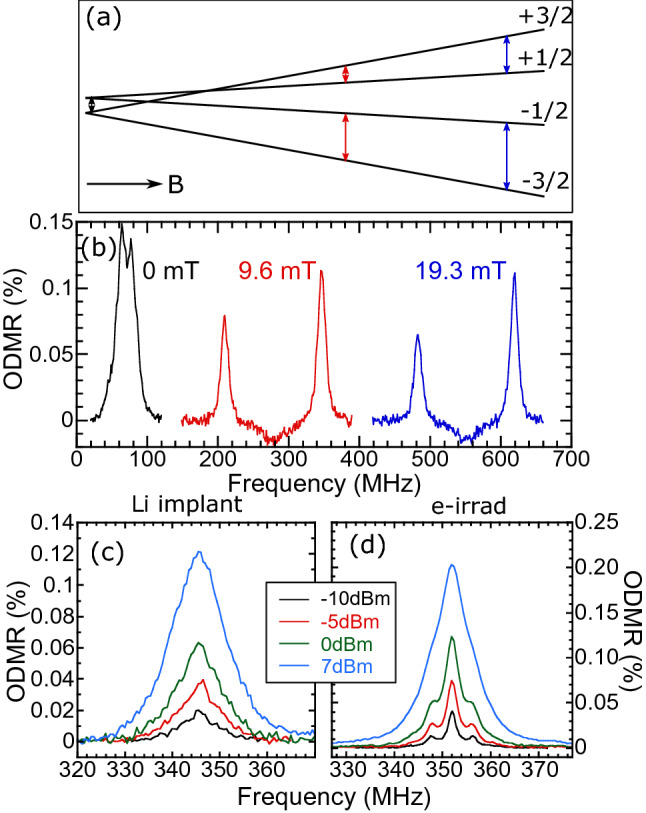
(**a**) Energies of the ground state S = 3/2 spin states of *V*2 as a function of magnetic field along the *c*-axis, with arrows representing the ODMR transitions. (**b**) Room temperature ODMR of 5 × 5 µm square implanted at 1000 ions/spot (2.01 × 10^14^ ions/cm^2^) taken at three different magnetic fields along the growth axis. The average RF power is 7 dBm. (**c**,**d**) Room temperature ODMR of the (**c**) Li^+^ implanted 5 µm × 5 µm square and (**d**) a reference 2 MeV electron-irradiated sample at about 10 mT for a series of RF powers.

Finally, in Fig. 4 we display RT ODMR spectra from an implant site. ODMR is measured before and after the anneal, with no clear differences. In this case we measure a large area 5 µm × 5 µm square implanted at 2 × 10^14^ ions cm^−2^, (same density as 1000 ions/spot) in order to obtain a large PL signal. The laser excitation spot is defocused in order to excite the entire square. Figure 4b plots ODMR for three different magnetic fields oriented along the growth axis. At zero applied field, there is a resonance centered at 70 MHz, with a small splitting between the two main lines, presumably due to a stray magnetic field. This zero field splitting matches that of the *V*2 defect. These lines correspond to the ${m}_{s}=\frac{1}{2}$ to ${m}_{s}=\frac{3}{2}$ transition and ${m}_{s}=-\frac{1}{2}$ to ${m}_{s}=-\frac{3}{2}$ transition. With an applied magnetic field, the two lines further split apart until the lower frequency transition ($+\frac{1}{2}\leftrightarrow +\frac{3}{2}$) crosses zero and both transitions increase, as illustrated in Fig. 4a. At the applied fields of 9.6 and 19.3 mT, the two lines are split by nearly double the zero-field splitting, as expected. The lower frequency transition is likely weaker due to proximity to anticrossings^19^. No ODMR from the *V*1 defect is observed, consistent with other RT measurements^15,19,46,47^. The origin of the weak, broad dip in between the two *V*2 ODMR lines is unknown.

Figure 4c,d display ODMR of the upper transition for the Li^+^ implant square and that of a reference *4H*-SiC epilayer sample, irradiated with 2 MeV electrons at a fluence of 3 × 10^18^ cm^−2^. At low radio frequency (RF) power, the hyperfine splitting of the transition^48^ due to next-nearest-neighbor ^29^Si can clearly be seen for the reference sample while it cannot be resolved for the Li^+^ implant sample. The maximum % ODMR is also weaker for the Li^+^ implant sample. This is due to the increased linewidth of the Li^+^ implant sample, which must be at least as large as the 4.3 MHz hyperfine splitting. For comparison, the linewidths of the peaks for the reference sample are 2.2 MHz (FWHM) at − 10 dBm. This broadening may be due to the high density of defects or due to inhomogeneity in the environment, perhaps from lattice damage. The origin of this broadening is an important topic for further study, particularly for applications in sensing. A rough estimate of the density of active ${V}_{Si}$, based on the estimated yield and SRIM calculations gives ~ 10^17^ cm^−3^, much higher than the expected density of ~ 10^16^ cm^−3^ for the electron irradiated sample^41^.

## Summary

We have demonstrated focused ion beam implantation of the lightest metal ions, Li^+^ into defect-free epitaxial 4*H*-SiC (0001) layers to form precisely located ${V}_{Si}$ emitter arrays. Fluorescence properties of the emitters are studied at low temperature and room temperature, using high-resolution scanning confocal microscopy. Our investigation revealed the presence of single defects at the lowest dose and a linear dependence of the fluorescence intensity on implantation dose over three orders of magnitude. ODMR measurements confirmed the presence of ${V}_{Si}$ spin ensembles as well. These high-density ensembles of Si vacancies (up to ~ 10^3^ per spot) localized within a small volume could be used for quantum sensing on the nano or micro scale.

## Methods

To determine the implantation fluence we measure the beam current using a built-in Faraday cup and a Keithley 6430 sub-femto-ammeter to determine the particle current (taking the charge state of the ion into account). The beam spot size is measured with a 80/20 fit to an etched Si grid structure. Using the particle current and the spot we then calculate the pulse length needed to achieve the desired fluence. For consistency the beam current is measured both before and after the implantation, and the average beam current is used in the calculation of the given ion beam fluence. We typically observe $\ll$ 10% variation in the beam current.

Cryogenic (4–40 K) and RT photoluminescence measurements are carried out by high-resolution scanning confocal fluorescence microscopy^8^. For LT measurements, the sample is mounted in a high NA (0.75) objective integrated closed-cycle optical cryostat with its growth axis oriented along the optical axis for face-on excitation and collection. This system is equipped with a fast steering mirror that is used to confocally raster scan/map the emission from the sample. It is capable of surveying patterned (${V}_{Si}$) emitters in the sample with a spatial resolution of ~ 600 nm in lateral (X–Y) direction. The continuous-wave off-resonant excitation (polarized horizontally on the sample, E┴*c*) of ≤ 1 mW (measured in front of the optical cryostat) is provided by a wavelength tunable Ti:Sapphire laser typically tuned to 745 nm (to eliminate the first- and second-order SiC Raman lines from the PL spectrum). The photoluminescence is collected by the objective and sent through an 850 nm long-pass filter to suppress the backscattered excitation laser and is then focused into a single mode fiber. Emission is then detected with one of the following: 1. a 750 mm length grating (150 g/mm) spectrometer (~ 0.35 nm resolution) along with a cooled Si CCD for recording the spectrum, 2. a silicon photon-counting avalanche photodiode (APD)/superconducting nanowire single photon detector (SNSPD) for detecting the integrated photon counts, or 3. a Hanbury Brown–Twiss (HBT) interferometry setup equipped with SNSPDs for the measurement of the second order autocorrelation function ${g}^{\left(2\right)}(\Delta t)$.

Some room temperature measurements are also performed with a higher (0.90) NA objective with the sample mounted on motorized stages. To correct for spatial drift during long maps and ${g}^{\left(2\right)}$ measurements, corrections were made periodically to position by using the spot in the lower left corner of Fig. 3a as a point of reference. ODMR is performed using this setup with a projected field electromagnet underneath the sample, giving a magnetic field along the growth axis (4° from the *c*-axis). An RF magnetic field for driving spin transitions is generated by a 50 µm diameter gold wire shorting a coaxial cable. The gold wire forms a half loop around the excitation spot with an approximate bend radius of 1–2 mm. Photoluminescence is sent directly to an InGaAs photodetector. The RF drive field is amplitude modulated at 700 Hz, and small changes to PL are measured with a lock-in amplifier while the RF frequency is scanned through the spin transitions.

## Supplementary Information

Supplementary Information
