# Supplementary material for: Arrays of Si vacancies in 4H-SiC produced by focused Li ion beam implantation
Source: Sci Rep. 2021 Feb 11;11:3561. doi: 10.1038/s41598-021-82832-x (PMC7878855; doi:10.1038/s41598-021-82832-x)
Supplement: Supplementary file 1 — Supplementary Information [file 41598_2021_82832_MOESM1_ESM.pdf]

Arrays of Si vacancies in 4*H*-SiC produced by focused Li ion beam implantation

Shojan P. Pavunny,[1*] Andrew L. Yeats,[2] Hunter B. Banks,[3] Edward Bielejec,[4] Rachael L. Myers-Ward,[2] Matthew T. DeJarld,[1] Allan S. Bracker,[2] D. Kurt Gaskill,[2†] and Samuel G. Carter[2*]

[1]ASEE Research Associate at the U. S. Naval Research Laboratory, 4555 Overlook Ave. SW, Washington DC 20375, USA

[2]U. S. Naval Research Laboratory, 4555 Overlook Ave. SW, Washington DC 20375, USA

[3]NRC Research Associate at the U. S. Naval Research Laboratory, 4555 Overlook Ave. SW, Washington DC 20375, USA

[4]Sandia National Laboratories, Albuquerque, New Mexico 87185, USA

†Current affiliation: Institute for Research in Electronics and Applied Physics, University of Maryland, College Park, MD, 20742, USA

Supplemental Material

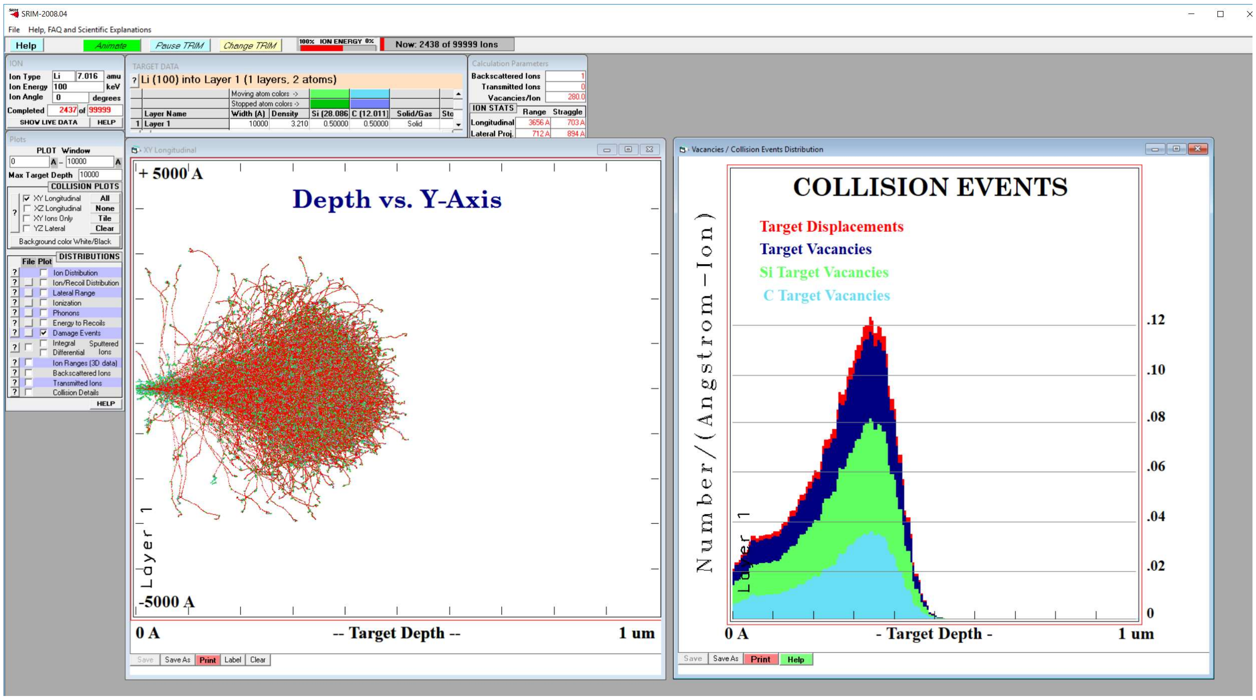

Fig. S1. SRIM simulation of the implantation of 100 keV Li ions into silicon carbide showing the plot of ion trajectories on the left and the vacancy distribution along the target depth on the right. (SRIM-2013 at http://www.srim.org/)

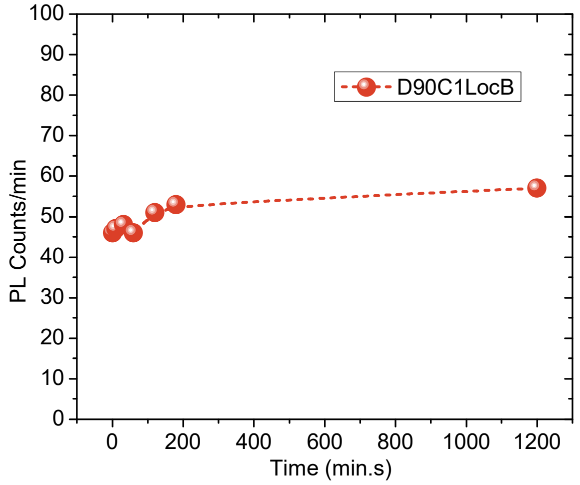

Fig. S2. Optical stability recorded on a particular spot at the 10 ions/spot implantation dose array.

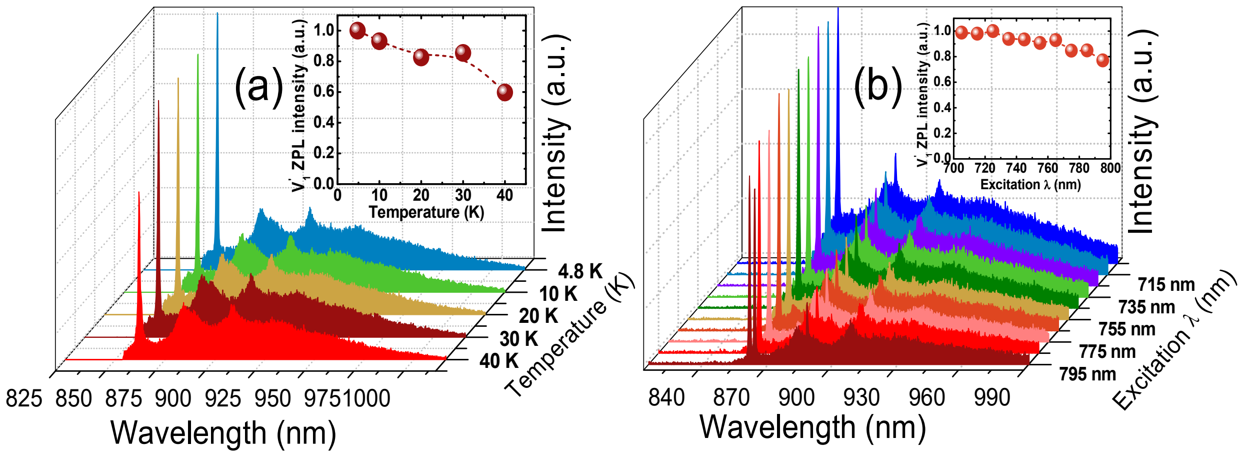

Fig. S3. (a) The sample temperature and (b) the excitation laser wavelength dependence of the emission spectrum at the lowest implantation dose of 10 ions/spot. The insets plot the $V1'$ ZPL intensity as a function of temperature and excitation wavelength.

We measure the dependence of the emission spectrum on temperature in Fig. S3(a) and excitation laser wavelength in Fig. S3(b) for the lowest implantation dose. Over a temperature range

of 4.8–40 K on a single spot, there is a small decrease in the $V1'$ emission intensity with the temperature rise, as shown in the inset of Fig. S3(a), but little change in the spectrum otherwise. In Fig. 3(b), no apparent change is observed in the fluorescence spectrum as the excitation wavelength is varied from 705 nm to 795 nm on a similar implantation spot, except for a gradual reduction of the $V1'$ ZPL brightness with increasing wavelength by ~20% (see inset). This could simply be due to the stronger absorption in the PSB at shorter wavelengths. One may note that the additional peak emerged at ~860 nm for 795 nm excitation is the LO (longitudinal optic phonon mode) Raman line (~950 cm^{-1}) of 4H-SiC.
